# Predictive Role of CX3CL1 and D-dimer Levels in Mortality Risk Stratification for COPD Patients With COVID-19

**DOI:** 10.7759/cureus.88782

**Published:** 2025-07-25

**Authors:** Jing Xue, Meimei Liu, Xin Liu, Na Gao, Wendong Hao

**Affiliations:** 1 Department of Internal Medicine, Jingbian County Hospital of Traditional Chinese Medicine, Yulin, CHN; 2 Department of Respiratory and Critical Care Medicine, Yulin Hospital, the First Affiliated Hospital of Xi'an Jiaotong University, Yulin, CHN

**Keywords:** copd: chronic obstructive pulmonary disease, covid-19, cx3cl1, d-dimer, mortality

## Abstract

Objective: In this retrospective study conducted at Yulin Hospital, the First Affiliated Hospital of Xi’an Jiaotong University (Yulin, SN, CHN), we aimed to investigate the predictive role of CX3CL1 and D-dimer for mortality in hospitalized chronic obstructive pulmonary disease (COPD) patients with COVID-19.

Methods: Complete blood counts and inflammatory cytokine levels were collected at admission from hospitalized COPD patients with COVID-19 to explore the relationship between inflammatory parameters and mortality of COPD patients with COVID-19.

Results: Compared to severe COPD with COVID-19 patients, circulating biomarkers of CX3CL1 (453.3 vs. 305.3 pg/mL,* *p<0.01) and D-dimer (1231.8 ng/mL vs. 680 ng/mL,* *p<0.01) were significantly elevated in critical illness participants. The C indices of inflammatory biomarkers CX3CL1 and D-dimer were 0.78 and 0.68, respectively. Furthermore, the prolonged illness of COPD patients with COVID-19 was associated with circulating inflammatory biomarkers of CX3CL1 (p=0.021) and D-dimer (p=0.041).

Conclusion: Circulating inflammatory biomarkers of CX3CL1 and D-dimer have shown the potential to predict mortality among COPD patients with severe COVID-19. Monitoring CX3CL1 and D-dimer levels could enhance clinical decision-making and risk stratification, potentially guiding more effective treatment strategies to improve patient outcomes.

## Introduction

The WHO declared COVID-19 a pandemic due to its large-scale global spread [1]. The clinical manifestations of COVID-19 vary greatly; the most common symptoms are fever, cough, sore throat, fatigue, etc., and in severe cases, life-threatening symptoms such as dyspnea may occur. The majority of COVID-19 patients have mild or no clinical symptoms; however, about 15% of patients have life-threatening disease, and this segment of cases has a high mortality rate [2]. COVID-19 disease progression and mortality are often associated with cytokine storms, cardiovascular disease, diabetes, malignancies, and chronic airway inflammatory diseases [3,4].

The cytokine storm is thought to be an important factor in the death of COVID-19 patients, a process that involves a large number of inflammatory mediators and immune cells. The CX3CR1 is a specific receptor for CX3CL1 and belongs to the chemokine receptor superfamily. The CX3CR1 is predominantly expressed on cytotoxic effector lymphocytes (including natural killer (NK) cells, cytotoxic T lymphocytes, and macrophages) and is a highly selective chemokine receptor and surface marker on cytotoxic effector lymphocytes, which are highly expressive of cellular granules such as perforin and granzyme B. The previous studies have shown that circulating CX3CL1 concentrations in chronic obstructive pulmonary disease (COPD) patients are enormously associated with emphysema severity, small airway obstruction, and airflow limitation severity [5-8]. However, studies on the association of circulating CX3CL1 levels with survival in COPD patients with COVID-19 have been rarely reported in the literature.

The D-dimer, a fibrin degradation product, is a relatively small protein fragment that is present in the blood after fibrinolysis degrades blood clots. The determination of serum D-dimer levels is a sensitive test for diagnosing pulmonary embolism and DIC in clinical practice [9,10]. Thus, elevated D-dimer levels of COVID-19 patients may help to rapidly identify patients with high disease severity, pulmonary complications, and venous thromboembolic risk in a prothrombotic state. This would assist with risk stratification and the early introduction of therapeutic measures that might reduce COVID-19-related morbidity and mortality.

Several clinical studies have shown that COPD patients have significantly elevated levels of D-dimer associated with hypercoagulability compared with controls [11-13]. However, there are limited reports on adverse clinical outcomes such as mortality and disease progression between D-dimer and COPD patients with COVID-19. Based on the above background, the present study investigated whether circulating inflammatory mediator CX3CL1 and D-dimer are associated with survival rates and prolonged illness among COPD patients with COVID-19.

## Materials and methods

Study design 

This is a retrospective study that was conducted at Yulin Hospital, the First Affiliated Hospital of Xi’an Jiaotong University (Yulin, SN, CHN), from January 1, 2023, to June 30, 2023. The study was conducted strictly per the Declaration of Helsinki and received approval from the Ethics Committee of Yulin Hospital, the First Affiliated Hospital of Xi'an Jiaotong University (approval no. 2023033). A total of 64 consecutive patients with severe or critical COVID-19 complicated by COPD were enrolled in the study. The 'severe' and 'critical' classifications were determined by WHO’s standardized definitions for COVID-19 illness. 'Severe' refers to cases meeting hypoxemia/respiratory compromise criteria (resting state without oxygen with peripheral oxygen saturation (SpO₂) ≤93%, tachypnea ≥30 breaths/min, or partial pressure of oxygen (PaO₂)/fraction of inspired oxygen (FiO₂) ≤300 mmHg) without requiring advanced organ support. 'Critical' specifically denotes those needing invasive mechanical ventilation and/or vasopressor support (indicating end-organ dysfunction or refractory respiratory failure). Eligibility required adults (≥18 years) with confirmed SARS-CoV-2 infection (via polymerase chain reaction (PCR) or serum antibodies), symptom onset within 10 days of enrollment, and pneumonia confirmed by imaging with evidence of rapid radiological progression [14].

Data collection

In this study, a variety of immune inflammatory parameters in COPD patients complicated with COVID-19 were collected. This included total bile acid, amylase, creatinine, D-dimer, WBC, lymphocytes, neutrophil-to-lymphocyte ratio (NLR), fibrinogen, cystatin C, and CX3CL1. In addition, general information such as age, gender, common symptoms, and comorbidities was also collected. 

In the morning, 5 ml of peripheral venous blood was collected from all subjects for experimental detection of immune inflammation parameters. Serum samples were obtained by centrifugation for 20 minutes at a speed of 3,000 revolutions per minute. Enzyme-linked immunosorbent assay (ELISA) from Shanghai Proton Biomedical Technology Co., Ltd. (Shanghai, HP, CHN) was used to detect the concentration level of immuno-inflammatory parameters. This was a retrospective study without blinding, and accordingly, the ELISA testing was not blinded to clinical outcomes to reduce measurement bias.

Statistical analysis

The collected data, such as immuno-inflammatory parameters and general patient information, were analyzed using the GraphPad Prism 5 (GraphPad Software, Inc., San Diego, CA, USA) and SPSS Statistics version 13.0 (IBM Corp., Armonk, NY, USA). All statistical tests were validated for normality using the Shapiro-Wilk test (significance level α=0.05). For data violating normality assumptions (p<0.05), the Mann-Whitney U nonparametric test was employed, with results annotated in the respective sections. The differences between different groups were processed using statistical methods such as the chi-square test or the Mann-Whitney U test. The receiver operating characteristic (ROC) curve was used to identify the optimal cut-off for D-dimer and CX3CL1 to predict disease severity. The p-values for the difference between survival rates were computed using Fisher's exact test. A p-value of < 0.05 was considered significant.

## Results

Basic characteristics of the subjects

This study is a retrospective study that enrolled a total of 64 COPD patients with COVID-19, including 38 women and 26 men, with 50 severe cases and 14 critical cases. The baseline clinical data of all participants included gender, age, symptoms, comorbidities, and clinical parameters. The age of the patients ranged from 39 to 87 years, with a median age of 60 years. The most common comorbidities were hypertension (13/64, 20.31%), diabetes mellitus (9/64, 14.06%), and coronary heart disease (7/64, 10.93%). The most common initial symptoms were cough (33/64, 51.56%), fever (25/64, 39.06%), and fatigue (12/64, 18.75%). The baseline characteristics of the subjects are shown in Table 1. 

**Table 1:** Characteristics of subjects at baseline Days and age are represented as mean ± SD, categorical variables are represented as number (%).

Characteristics		No. of patients (total n=64）	Severe illness （n=50）	Critical illness （n=14）	Chi-square test	p-value
Demographics	Male	24 (37.50%)	19 (38.00%)	5 (35.71%)	0.21	> 0.05
	Female	40 (62.50%)	31 (62.00%)	9 (64.29%)		
	Age (years)	60±12.0	62±10.3	73±9.8	7.15	0.047
Symptoms and signs	Fever	25 (39.06%)	18 (36.00%)	6 (42.85%)	6.41	> 0.05
	Fatigue	12 (18.75%)	8 (16.00%)	3 (21.43%)	0.17	> 0.05
	Cough	33 (51.56%)	24 (48.00%)	7 (50.00%)	0.03	> 0.05
	Anorexia	9 (14.06%)	7 (14.00%)	2 (14.28%)	3.82	> 0.05
	Diarrhea	7 (10.93%)	6 (12.00%)	1 (7.14%)	0.22	> 0.05
	No-symptom	6 (9.37%)	5 (10.00%)	1 (7.14%)	2.7	> 0.05
Clinical parameters	Days from onset to discharge	38.16±19.0	37.51 ±18.3	33.48±14.0	3.35	> 0.05
	Days from onset to admission	18.63±13.5	18.38±13.3	17.14±11.8	5.36	> 0.05
	Discharged	57 (89.06%)	48 (96.00%)	9 (64.28%)	9.64	0.022
	Died	6 (9.37%)	1 (2.00%)	5 (35.71%)	28.17	0.017
Comorbidity	Pulmonary embolism	4 (6.25%)	2 (4.00%)	2 (14.28%)	7.88	0.037
	Asthma	3 (4.68%)	2 (4.00%)	1 (7.14%)	1.19	> 0.05
	Hypertension	13 (20.31%)	9 (18.00%)	3 (21.43%)	4.25	> 0.05
	Coronary heart disease	7 (10.93%)	4 (8.00%)	2 (14.28%)	2.8	> 0.05
	Diabetes	9 (14.06%)	6 (12.00%)	4 (28.57%)	32.78	0.014
	Stroke	1 (1.56%)	1 (2.00%)	0 (0%)	0.09	> 0.05
	Malignant tumor	2 (3.12%)	1 (2.00%)	1 (7.14%)	5.47	> 0.05

A stark contrast in mortality rates was observed between severe illness and critical illness (p=0.017). The severe COVID-19 group demonstrated 2.00% mortality (1/50), while the critical group showed significantly higher mortality at 35.71% (5/14), representing a 17.9-fold increased risk. This significant difference underscores the prognostic value of initial severity stratification for clinical outcomes. The mortality pattern aligns with established COVID-19 severity paradigms observed in other populations.

Laboratory findings of participants with different illness severities

We examined multiple immuno-inflammatory parameters and compared their differences in participants with different disease severity. The circulating CX3CL1 and D-dimer expression levels in patients with severe illness were 305.3 (123.8-567.5) pg/mL and 680 (365.9-1420) ng/mL, respectively. Serum CX3CL1 and D-dimer levels were 453.3 (229.6-813.7) pg/mL and 1231.8 (607-3306.2) ng/mL in critical illness subjects, respectively. There was a major difference in the levels of CX3CL1 and D-dimer between severe and critically ill patients p<0.01. In addition, the NLR (12.64, p=0.027), C-reactive protein (105.6 mg/L, p=0.044), cystatin C (0.99, p=0.045), WBC (13.46×109/L, p=0.034), and neutrophils (10.24×109/L, p=0.021) were significantly higher than those in patients with severe illness. Circulating immuno-inflammatory parameter concentration levels are given in Table 2.

**Table 2:** Immuno-inflammatory parameters in different illness severity All experimental data were represented by median (range), and the p-values were calculated using the Mann-Whitney U test.

Inflammatory parameters	No. of patients (total n=64)	Severe illness (n=50)	Critical illness (n=14)	Mann-Whitney U test	p-value
Total bile acid (μmol/L)	3.29 (2.11-4.19)	3.22 (1.98-4.36)	3.41 (2.93-4.74)	420.0	> 0.05
Amylase (U/L)	58.00 (37.91-78.84)	59.00 (37.00-79.44)	65.21 (61.49-83.30)	398.5	> 0.05
Creatinine (mol/L)	61.82 (53.16-75.38)	68.31 (55.96-79.36)	73.31 (59.86-88.97)	332.8	> 0.05
Fibrinogen (g/L)	2.61 (2.17-3.43)	2.85 (2.22-3.49)	3.32 (2.84-3.78)	402.5	> 0.05
Cystatin C ( mg/L)	0.92 (0.81-1.10)	0.91 (0.78-1.01)	0.99 (0.82-1.15)	312.0	0.045
White blood cell count (10^9^/L)	7.59 (2.36-21.81)	7.15 (2.39-14.70)	13.46 (3.51-21.72)	282.5	0.034
Neutrophils (10^9^/L)	4.51 (1.40-19.61)	4.05 (1.39-12.88)	10.24 (2.59-19.68)	254.9	0.021
Lymphocytes (10^9^/L)	1.45 (0.36-5.58)	1.42 (0.41-3.92)	1.63 (0.38-5.67)	349.7	> 0.05
Neutrophil-to-lymphocyte ratio	4.60 (0.77-28.41)	3.70 (0.78-27.23)	12.64 (1.56-22.47)	271.8	0.027
Platelet count (10^9^/L)	239.17 (47-651)	262.95 (48-639)	195.81 (106-374)	355.3	> 0.05
D-dimer (ng/mL)	835 (365.9-3306.2)	680 (365.9-1420)	1231.8 (607-3306.2)	226.7	< 0.01
C-reactive protein (mg/L)	89.61 (43.9-213.7)	76.5 (42.9-177.2)	105.6 (62.4-211.5)	305.1	0.044
Erythrocyte sedimentation rate (mm/h)	30.17 (24.86-46.60)	35.00 (27.61-53.64)	39.05( 32.18-60.25)	379.0	> 0.05
CX3CL1 ( pg/mL)	349.2 (123.8-814.7)	305.3 (123.8-567.5)	453.3 (229.6-813.7)	205.5	< 0.01

Association of biomarkers with in-hospital mortality

**Figure 1:**
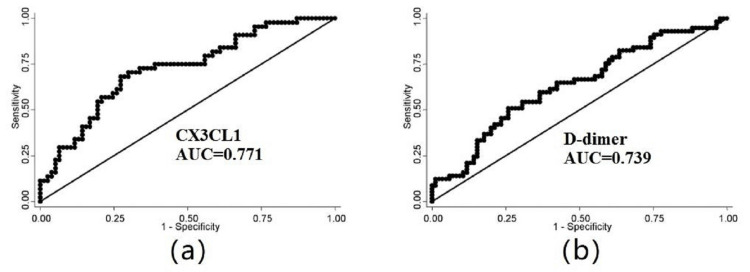
The ROC curve for biomarkers to predict mortality ROC: Receiver operating characteristic, AUC: Area under the curve

The ROC curve was plotted, and the cut-off values for the two immuno-inflammatory parameters were obtained. The cut-off values for CX3CL1 and D-dimer were 433 ng/mL (sensitivity 84.7%, specificity 76.2%; Figure 1a) and 764 ng/L (sensitivity 66.1%, specificity 58.6%; Figure 1b), respectively. Moreover, the C-indices of CX3CL1 and D-dimer were 0.78 (95% CI: 0.71-0.86) and 0.68 (95% CI: 0.58-0.78), respectively. The CX3CL1 predicts better in-hospital mortality in COPD patients with COVID-19. The C-indices for CX3CL1 and D-dimer are shown in Table 3.

**Table 3:** The C-statistic of immuno-inflammatory parameters to predict mortality.

Predictor cytokines	C-index	95% CI
CX3CL1	0.78	0.71-0.86
D-dimer	0.68	0.58-0.78

Relationship between biomarkers and duration of illness

**Figure 2:**
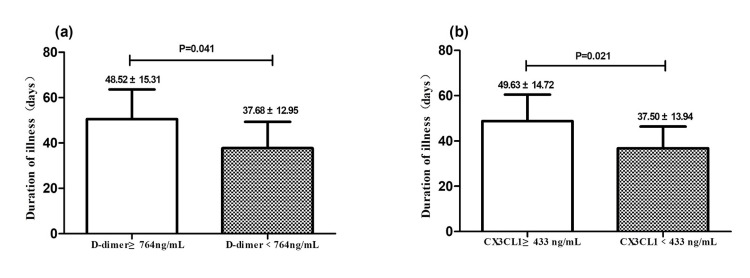
Association between immuno-inflammatory parameters and duration of illness The duration of illness in all participants was stratified by the level of D-dimer (a) and CX3CL1 (b). The p-value was calculated by the statistical method of Wilcoxon's rank-sum test.

Chronic obstructive pulmonary disease patients with COVID-19 exhibiting high D-dimer levels (above 764 ng/L) were found to have a significantly longer duration of illness compared to those with lower D-dimer levels (below 764 ng/L). The data showed that patients with high D-dimer levels experienced an average illness duration of 48.52±15.31 days, while those with lower levels had an average duration of 37.68±12.95 days. This difference was statistically significant with a p-value of 0.041, indicating a strong association between elevated D-dimer levels and extended illness duration (Figure 2a).

Similarly, high levels of CX3CL1 (above 433 ng/mL) were also associated with a longer duration of illness in COPD patients with COVID-19. Patients with elevated CX3CL1 levels experienced an average illness duration of 49.63±14.72 days, compared to 37.50±13.94 days for those with lower CX3CL1 levels. All patients in this study received glucocorticoid therapy, with serial monitoring of CX3CL1 and D-dimer levels. Post-treatment measurements showed a decline in both biomarkers compared to baseline admission levels, suggesting their potential utility as indicators of treatment response. Although four critically ill patients with interstitial lung disease (ILD) received adjusted steroid dosing (methylprednisolone escalated from 40-80 mg/day to 160-500 mg/day), the small sample size (n=4) precluded statistical confirmation of whether intensified therapy shortened disease duration. Future studies with larger cohorts are warranted to validate these observations. This association was also statistically significant, with a p-value of 0.021, reinforcing the link between higher inflammatory marker levels and prolonged illness (Figure 2b).

## Discussion

The ongoing COVID-19 pandemic has significantly impacted patients with COPD, particularly those experiencing severe cases of the virus. In this context, our current study focuses on exploring the association between various inflammatory biomarkers and clinical features among COPD patients with severe COVID-19 in Yulin, Northwest China. Our findings indicate that specific laboratory variables are closely linked to the severity, mortality, and duration of illness in this patient population.

Our in-depth analysis reveals significant alterations in immune indicators and presents strong evidence supporting the use of specific biomarkers for prognostic evaluation. These findings highlight the significant immune disturbances in COPD patients with severe COVID-19 and reinforce the utility of specific biomarkers in predicting clinical outcomes. The observed increase in WBC, neutrophil count, NLR, and C-reactive protein levels in critically ill patients is consistent with previous research, underscoring the importance of these indicators in assessing disease severity [15]. Furthermore, the strong association between elevated D-dimer and CX3CL1 levels and increased mortality provides valuable insights for clinicians. These biomarkers can be used to identify patients at higher risk of poor outcomes, facilitating early and targeted interventions. The combined analysis of D-dimer and CX3CL1 levels, in particular, offers a powerful tool for mortality prediction, enabling more accurate risk stratification.

The ability of SARS-CoV-2 to infect and damage multiple organ systems can lead to systemic inflammation, widespread tissue damage, and multi-organ failure, which is often life-threatening [16]. Cystatin C has garnered significant attention in the medical community due to its role as an inflammatory biomarker. Its importance has been highlighted in various studies, particularly in the context of COVID-19 [17], renal dysfunction [18], diabetes [19], and cardiovascular disease [20]. Recent studies, including those by Zinellu et al., have highlighted the significance of elevated cystatin C levels in critical COVID-19 patients. This systematic review explores the findings of these studies, focusing on the implications of cystatin C as a predictor of disease severity and mortality [21]. Our research corroborates the findings of Zinellu et al., reinforcing the importance of cystatin C as a biomarker in COVID-19. The current data indicate that cystatin C levels are significantly higher in patients with critical illness compared to those with severe COVID-19, suggesting its role in predicting adverse outcomes. 

The CX3CL1, also known as fractalkine, is a chemokine involved in the adhesion and migration of leukocytes. It plays a crucial role in the inflammatory response and has been identified as a significant predictor of disease outcomes in COPD patients with COVID-19. The current research has also revealed a link between duration of illness and circulating CX3CL1 concentrations. CX3CL1 plays a crucial role in recruiting CX3CR1+ monocytes and cytotoxic T cells to sites of inflammation, thus contributing to the development of an inflammatory environment [22]. The recruitment of CX3CR1+ monocytes and cytotoxic T cells can lead to an inflammatory environment, a phenomenon observed in conditions like atherosclerosis [23]. In addition, studies on animal models, particularly rats, have demonstrated that increased levels of CX3CL1 can lead to thrombosis [24].

The COVID-19 pandemic has brought new insights into the role of chemokines in viral infections. SARS-CoV-2, the virus responsible for COVID-19, penetrates endothelial cells and inhibits the expression of ACE2 receptors. This inhibition causes an imbalance in the renin-angiotensin system (RAS), leading to endothelial damage and contributing to thrombosis. The relationship between CX3CL1 and endothelial damage in COVID-19 patients suggests that elevated CX3CL1 levels could exacerbate thrombotic events [25]. Research conducted by Tang et al. has shown that heparin, an anticoagulant, provides favorable outcomes in the prevention and treatment of thrombosis in COVID-19 patients. Heparin's effectiveness is partly due to its ability to decrease CX3CL1 transcription and protein levels in human umbilical cord endothelial cells stimulated by IFN-γ. This reduction in CX3CL1 levels subsequently decreases monocyte adhesion, thereby mitigating inflammation and thrombosis [26,27]. Thrombosis can lead to damage in multiple organs, including the liver, heart, and kidneys. This organ damage can be correlated with the duration and severity of diseases involving thrombotic complications. The ability to detect soluble CX3CL1 in serum makes it a potential prognostic marker for thrombotic complications. Monitoring CX3CL1 levels could help identify COVID-19 patients who require more aggressive antithrombotic treatment, thereby improving clinical outcomes.

Among these clinical markers, D-dimer levels have emerged as a significant predictor of mortality and disease progression. Increased D-dimer levels during the course of COVID-19 indicate a higher likelihood of the disease progressing to a severe form, thereby significantly increasing the risk of mortality [28]. One study demonstrated that each 1 µg/mL increase in D-dimer levels at the time of admission correlates with a 6% increase in the risk of death and an 8% increase in the likelihood of requiring mechanical ventilation [29]. Another study highlighted that D-dimer levels higher than 2 µg/mL at admission can serve as a predictor of mortality during hospitalization. Observing a D-dimer value exceeding this threshold at any point during hospitalization is a strong indicator of a poor prognosis. Therefore, elevated D-dimer levels are a powerful prognostic marker in COPD patients with COVID-19, providing valuable information on the likelihood of disease progression and mortality. The absence of multivariable adjustment for key confounders of age and comorbidities raises concerns about potential bias in the findings. Although further multicenter confirmation is needed, the association of D-dimer fluctuations with disease severity grades suggests its provisional utility as a monitoring biomarker.

Our study further explored the association between disease duration and circulating concentrations of D-dimer. Patients with elevated D-dimer levels require a longer recovery time compared with those with low D-dimer levels. Inflammation and hemostasis are closely linked in the body’s pathophysiological processes. When the body is in a state of inflammatory response, the coagulation cascade system is locally activated by inflammatory mediators and pro-inflammatory factors to limit and converge inflammation [30]. Inflammatory mediators promote the expression of vascular endothelial cell-related tissue factors, leading to increased fibrin degradation and higher D-dimer levels. Disruptions in the coagulation system may induce the blood vessel endothelium to produce more cytokines, triggering an inflammatory storm that can lead to multiple organ failure, including the liver, kidneys, and heart. These mechanisms explain the correlation between elevated D-dimer levels and prolonged disease duration.

Our research has several potential limitations. First, the study's relatively small sample size may limit the generalizability of the findings. Additionally, the lack of multivariable adjustment for potential confounders, such as age or comorbidities, could further affect the reliability of the observed associations. We recruited participants who met both COPD and COVID-19 diagnostic criteria. Moreover, all participants enrolled in our study were patients with severe or critical COVID-19. So there are fewer included cases. The observed trends are hypothesis-generating and warrant validation in larger, multicenter cohorts, with an overall understated confidence given the small sample size. Second, some crucial parameters, such as PaO2, PaCO2, pH, and SaO2, were not included in the study due to their absence in the health information system. Third, this research is a single-center retrospective study. Fourth, the observed elevation in CX3CL1 and D-dimer levels in critical cases may have been partially attributable to concurrent bacterial infections rather than COVID-19 severity alone.​​ ​​

Further large-scale studies with comprehensive infection screening are warranted to better elucidate the independent predictive value of these biomarkers.​ To enhance the robustness and generalizability of the results, future studies should aim to collect immunological parameters from COPD patients with COVID-19 across multiple centers. This would provide more convincing evidence regarding the prediction of inflammatory biomarkers related to disease severity and mortality in this patient population.

## Conclusions

The findings of this study underscore the critical role of systemic inflammation in modulating disease trajectory among COPD patients with COVID-19. Elevated circulating levels of CX3CL1 and D-dimer not only serve as independent predictors of mortality but also correlate with prolonged hospitalization, highlighting their dual utility as prognostic biomarkers and therapeutic targets. This proposed mechanism, supported by prior literature, suggests that CX3CL1-mediated immune cell recruitment may amplify pulmonary damage through excessive inflammation, while elevated D-dimer reflects COVID-19-associated coagulopathy driving thrombosis. However, these relationships are derived from existing studies rather than being directly demonstrated in the current research. The combined assessment of these biomarkers could enable stratification of patients into distinct risk categories, allowing clinicians to tailor interventions such as anticoagulation therapy or immunomodulatory agents to mitigate disease progression.
